# D, L-Buthionine-(S, R)-sulfoximine recapitulates the anti-obesity effects of sulfur amino acid restriction without the associated deleterious effects on bone in male mice

**DOI:** 10.18632/aging.206358

**Published:** 2026-03-01

**Authors:** Naidu B. Ommi, Dwight A. L. Mattocks, Mark C. Horowitz, Sailendra N. Nichenametla

**Affiliations:** 1 Orentreich Foundation for the Advancement of Science Inc., Cold Spring-on-Hudson, NY 10520, USA; 2 Department of Orthopaedics and Rehabilitation, Yale University School of Medicine, New Haven, CT 06510, USA

**Keywords:** bone, aging, methionine, glutathione, redox

## Abstract

Sulfur amino acid restriction (SAAR), a diet low in methionine and lacking cysteine, reduces obesity but also lowers bone mineral density (BMD) and increases marrow adipose tissue. Because the SAAR diet lacks cysteine, it exerts cysteine restriction (CysR), in addition to methionine restriction (MetR). We previously reported that the anti-obesity effect of the SAAR diet was exclusively due to CysR. Follow-up studies revealed that CysR decreases obesity by lowering glutathione (GSH), and that D, L-buthionine-(S, R)-sulfoximine (BSO), an inhibitor of GSH biosynthesis, recapitulates the SAAR-induced lean phenotype on a methionine-replete diet. Here, we investigated whether the detrimental effects of the SAAR diet on bone are mediated solely by CysR and whether BSO, similar to the SAAR diet, exerts deleterious effects. Male obese C57BL6/NTac mice were fed high-fat diets with 0.86% methionine (control diet, CD), 0.12% methionine (SAAR diet), SAAR diet supplemented with a GSH precursor, N-acetylcysteine (NAC) in water, and CD supplemented with BSO in water. Femurs and tibiae of SAAR mice had lower trabecular and cortical BMD, fewer osteoblasts, reduced biomechanical strength, and more marrow adipocytes than in CD mice. NAC reversed all these effects, suggesting that CysR mediates the detrimental effects of the SAAR diet on bone. Despite its anti-obesity effects, BSO did not exert any detrimental effects on bones. Future studies should investigate mechanisms, age-at-onset, tissue-specific, and gender-specific effects of BSO on bone health. Long-term studies to establish the therapeutic efficacy and off-target effects of BSO are critical for developing it as an anti-obesity drug in humans.

## INTRODUCTION

Due to trade-offs in life-history traits, lifespan- and healthspan-extending laboratory interventions often have some deleterious consequences [1, 2]. Identifying the mechanisms and causal factors underlying these effects provides an opportunity to modify the interventions, ward off negative consequences, and selectively induce beneficial effects using drugs. Sulfur amino acid restriction (SAAR), lowering the dietary concentration of sulfur amino acid, methionine (Met), and eliminating cysteine (Cys), extends lifespan by up to 45% in various laboratory models, including rodents [3]. A remarkable metabolic benefit of the SAAR diet in rodents is resistance to obesity even on an *ad libitum* high-fat diet (60% Kcal from fat) and a rapid (3–4 weeks) decrease in body fat in obese mice [4, 5]. Concomitant to this robust decrease in peripheral adipose depot weights and total fat mass, the SAAR diet increases bone marrow adipocytes while decreasing bone mineral density and biomechanical strength [6, 7]. The mechanisms responsible for these adverse effects on bone strength and marrow adipocytes are only partially characterized.

Met and Cys are two proteinogenic sulfur amino acids essential for normal health and metabolism. Animal cells cannot synthesize Met and thus must obtain it through diet, while Cys can be synthesized from Met. Due to its dispensable nature, most studies with the SAAR diet eliminated Cys from their diet formulations. Because Cys is metabolically essential, rodents on the SAAR diet synthesize some of it from Met, but cannot achieve the same high tissue concentrations as those on the control diets [8]. Thus, rodents on the SAAR diet undergo both Met restriction (MetR) and Cys restriction (CysR). Whether the anti-obesity effects of the SAAR diet in mice are due to MetR or CysR remained unknown until recently. We just reported that eliminating CysR through supplementation with N-acetylcysteine (NAC), a precursor of Cys, reversed the anti-obesity effects of the SAAR diet [9]. This study suggests that CysR, but not MetR, is responsible for the anti-obesity effects of the SAAR diet. Whether CysR is also accountable for the SAAR diet-induced increase in marrow adipocyte numbers and decrease in bone mineral density (BMD) remains unknown.

Cys is stored in cells as glutathione (GSH), a tripeptide of glutamic acid, Cys, and glycine. While it is well-known for its anti-oxidant and detoxification roles, GSH is essential for multiple other functions, including cell signaling [10, 11]. Considerable evidence from the literature shows that GSH plays a vital role in bone formation [12]. Runx2 is a transcription factor that commits skeletal stem cells to the osteoblast lineage. *Runx2^+/−^* haplo-insufficient mice suffer from impaired embryonic bone development [13]. Supplementing these mice with NAC, a precursor of Cys and thus of GSH, improved bone development [13]. Another study reported that the bone loss induced by estrogen deficiency in ovariectomized mice was prevented by NAC supplementation [14]. These studies provide strong evidence for the mechanistic role played by GSH in bone formation and development. In animals on the SAAR diets, GSH concentrations in the liver and kidney - tissues where it is most abundant - are reported to decrease to 20% to 69% of the normal levels [9]. In a previous study, we demonstrated that pharmacological lowering of GSH by using the inhibitor of its biosynthesis, D, L-buthionine-(S, R)-sulfoximine (BSO), exerts similar anti-obesity effects as the SAAR diet [9]. To conduct clinical trials with BSO as an anti-obesity intervention, it is essential to determine whether it exerts detrimental effects on bone, similar to those observed with the SAAR diet. However, no such study has been conducted. In this study, we investigated whether NAC supplementation reverses the detrimental effects of the SAAR diet on bone and whether pharmacological inhibition of GSH biosynthesis by BSO recapitulates the SAAR diet-induced deleterious impact on bones.

## MATERIALS AND METHODS

### Animal husbandry

All animal procedures were conducted according to the Institutional Animal Care and Use Committee guidelines of the Orentreich Foundation for the Advancement of Science Inc. The biological specimens required for the current study were collected from the same animals used in a recently published study [9]; no additional animals were used. Animal husbandry, dietary formulations, drug concentrations, and administration procedures are detailed elsewhere [9].

Briefly, four groups of 18-week-old male diet-induced obese C57BL6/NTac mice (Model No. DIO-B6-M, Taconic Biosciences, Germantown, NY, USA) were fed a high-fat diet (60% Kcal from fat) with two concentrations of Met. The control diet group (CD) received a diet with 0.86% w/w Met without Cys. The SAAR group received a diet with 0.12% w/w Met without Cys. The third group (NAC) also received the same diet as the SAAR group, but was supplemented with 30 mM NAC in water (Catalog No. A9165, Sigma Aldrich, St. Louis, MO, USA). The fourth group (BSO) received the same diet as the CD group, plus 30 mM BSO (Catalog No. B690250, Toronto Research Chemicals Inc., Toronto, ON, Canada) dissolved in water, which inhibits GSH biosynthesis. After treating mice with diets and drugs for 13 weeks, soft tissues and bones were collected.

### Preparation of long bones

After isolating and stripping the soft tissue, the left tibiae were fixed overnight in 70% ethanol and used for micro-computed tomography (micro-CT) analyses. Following micro-CT, the same tibiae were prepared for histologic analyses using non-decalcified thin sections (5 μm) stained with toluidine blue as described elsewhere [15]. The right tibiae were fixed in 10% neutral-buffered formalin for 24 hours, rinsed in water, decalcified in 4% EDTA for 20 days, and used to measure bone marrow adipocytes. Left femurs were wrapped in phosphate-buffered saline-soaked gauze and stored at 4°C for mechanical testing.

### Micro-CT

Femurs and tibiae were analyzed by micro-CT using a Scanco micro-CT-35 (Scanco Medical; Bruttisellen, Switzerland) at a maximum voxel size of 10 μm, an integration time of 500 ms, and an energy of 55 kVp. Reconstructions of the micro-CT data were generated with AltaViewer software, version 1.1.2 (Numira Biosciences; Salt Lake City, UT, USA). Trabecular bone measurements include bone volume (BV, mm^3^), total volume (TV, mm^3^), BV/TV, connective density (Conn-dens, 1/mm^3^), trabecular number (Tb.N, 1/mm), trabecular spacing (Tb.Sp, mm), and trabecular thickness (Tb.Th, mm). Cortical bone measurements include cortical thickness (Ct.Th, mm), endosteal radius (mm), periosteal radius (mm), endosteal circumference (mm), periosteal circumference (mm), and polar moment of inertia (pMOI, mm^4^).

### Histomorphometry

Static histomorphometry was performed on left tibiae, as previously described [15]. Briefly, microscopic analysis of static parameters was performed using an Olympus microscope and the Osteomeasure system (Osteometrics; Atlanta, GA, USA). Calculations of various parameters were based on the methods of Parfitt et al. [16]. All parameters were measured on trabecular bone beginning just under the growth plate in the primary spongiosa, excluding endosteal surfaces. The field size was 350 μm, and the area measured under the growth plate extended to 700 μm, where most of the trabecular bone is located.

### Measurement of marrow adipocytes

Formalin-fixed, decalcified tibiae were stained for neutral lipids using a 1:1 mixture of 2% aqueous osmium tetroxide and 5% potassium dichromate for 48 hours, as previously described [17]. Bones were then washed in tap water and imaged by micro-CT in water with an energy of 55 kVp, an integration time of 500 ms, and a maximum voxel size of 10 μm (i.e., the “high” resolution setting on a Scanco microCT-35 instrument). The selection of volumes of interest (VOIs) was performed to facilitate the visualization and quantification of marrow adipocytes. The results are presented as volumetric measurements, similar to the volumetric bone measurement, bone volume/total volume (BV/TV).

### Biomechanical testing

Femurs were loaded to failure using a four-point bending test. Tests were conducted by loading the femur in the anterior–posterior orientation, such that the posterior quadrant was subjected to tensile loads. The widths of the upper and lower supports of the four-point bending apparatus were 3 and 7 mm, respectively. Tests were conducted with a deflection rate of 0.05 mm/sec using a servohydraulic Instron model 8874 testing machine (Instron Corp.; Norwood, MA, USA). The load and mid-span deflection measurements were acquired at a sampling frequency of 200 Hz. Load deflection curves were analyzed for stiffness, maximum load, and total work to fracture. Yield was defined as a 10% reduction in the secant stiffness relative to the initial tangent stiffness and used to determine the post-yield deflection, which was defined as the deflection at failure minus the deflection at yield. Tests were performed at room temperature, and bones were kept moist with PBS.

### Osteocalcin quantification

Plasma osteocalcin concentrations were quantified using an ELISA kit from Novus Biologicals (Catalog No: NBP2-68151) following the manufacturer’s recommendations.

### Statistical analysis

GraphPad Prism was used for statistical analysis. All data were first analyzed using a one-way analysis of variance, followed by a post-hoc test (Tukey’s multiple comparison test) to find pair-wise differences. Occasionally, a two-tailed Student’s *t*-test was used if the differences in the means of two groups were greater than 25% and the Tukey’s test failed to detect these differences. We employed this approach to provide the reader with more information, and *P*-values from both Tukey’s test (n.s., not significant) and *t*-tests (#) were included in the figures. Pair-wise differences were considered statistically significant if *P* < 0.05 and were reported by asterisks (^*^ for *P* ≤ 0.05, ^**^ for *P* ≤ 0.01, ^***^ for *P* ≤ 0.001, and ^****^ for *P* ≤ 0.0001). The sample size was six to seven per group. Although pairwise comparisons were performed between all groups, only four comparisons relevant to our hypothesis are shown in the figures (CD vs. SAAR, CD vs. NAC, CD vs. BSO, and SAAR vs. NAC).

## RESULTS

### NAC reverses the SAAR diet-induced changes in body composition

It has been reported that Cys supplementation reverses SAAR diet-induced effects, including changes in plasma hormone concentrations (IGF-1, FGF-21, leptin, and adiponectin), a decrease in body weights, and epididymal adipose depot weights in rodents fed diets with normal fat content; it was unknown whether the same effects could be recapitulated by feeding a high-fat diet [18, 19]. Similar body weights (Supplementary Figure 1A) and total fat mass (Supplementary Figure 1B) without loss of lean mass (Supplementary Figure 1C) between the CD and NAC groups demonstrate that the reversal occurs when feeding on a high-fat diet too (Supplementary Figure 1). Although the effects of BSO on body weights, total fat mass (Supplementary Figure 1B), and epididymal adipose depot weights (Supplementary Figure 1D) were not as pronounced as the changes induced by the SAAR diet, they were in the same direction. But unlike the SAAR diet, BSO did not decrease lean mass (Supplementary Figure 1C). These data indicate that CysR, but not MetR, is causally associated with the anti-obesity effects of the SAAR diet (Supplementary Figure 1).

### Unlike the SAAR diet, BSO does not decrease trabecular and cortical bone mineral density

Two factors determine bone strength: bone mineral content (also known as bone mineral density) and bone microarchitecture, which refers to the physical arrangement of the material in cortical bone and the arrangement and density of the trabecular struts (rods and plates) at the microscopic level. Mice on the SAAR diet had less trabecular bone than those on the CD diet (Figure 1A). Several measures of trabecular bone, including the ratio of bone volume to total volume (BV/TV, Figure 1B, 44% of CD, *P* < 0.001), connectivity density (Figure 1C, 20% of CD, *P* < 0.001), and trabecular number (Figure 1D, 71% of CD, *P* < 0.001) were lower in the SAAR mice. Trabecular space, which is inversely associated with trabecular number, was higher in the SAAR mice (Figure 1E, 141% of CD, *P* < 0.0001). NAC reversed all these indicators to the levels observed in the CD mice; importantly, BSO had no effect. Trabecular thickness was similar in all groups (Figure 1F).

**Figure 1:**
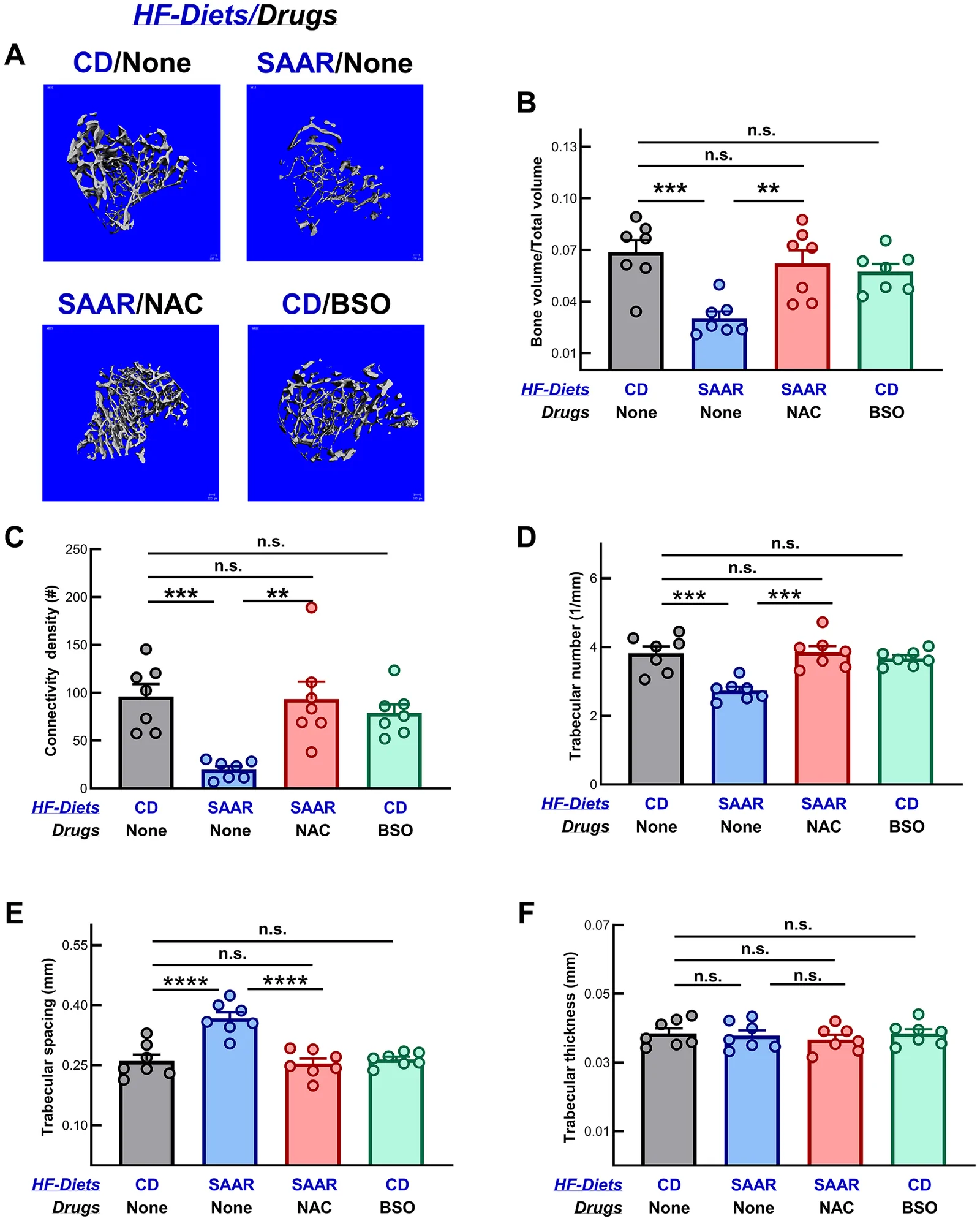
**Unlike the SAAR diet, BSO does not decrease trabecular bone mineral density.** (**A**) Representative images of the micro-CT scanning of trabecular bone in tibiae show that the SAAR diet lowered multiple measures of trabecular bone density, including (**B**) the ratio of bone volume to total volume (SAAR < CD), (**C**) connectivity density (SAAR < CD), and (**D**) trabecular number (SAAR < CD). However, the SAAR diet increased the (**E**) trabecular space (SAAR > CD) but did not affect (**F**) trabecular thickness (SAAR = CD). As we hypothesized, NAC reversed all the SAAR-induced changes to the levels observed in the CD group, while BSO did not affect any of these parameters (SAAR < [CD = NAC] in figures B, C and D; SAAR > [NAC = CD] in figure E). Note: One-way ANOVA followed by Tukey’s multiple comparison tests was used to find group-wise differences (^*^P ≤ 0.05, ^**^P ≤ 0.01, ^***^P ≤ 0.001, ^****^P ≤ 0.0001; bars and error bars represent means and standard errors of the mean; *n* = 7/group).

The cortical thickness of femurs in mice on the SAAR diet was 84% of that in the CD mice (Figure 2A, 2B, *P* < 0.0001), while the periosteal radius was 134% (Figure 2C, *P* < 0.05). However, in NAC mice, the thickness and radius were similar to those in the CD mice, indicating that the underlying cause of the detrimental effects of the SAAR diet on the bone is CysR, and not MetR. None of the three interventions, i.e., SAAR, NAC, and BSO, changed the endosteal radius and bone tissue density (Figure 2D, 2E).

**Figure 2:**
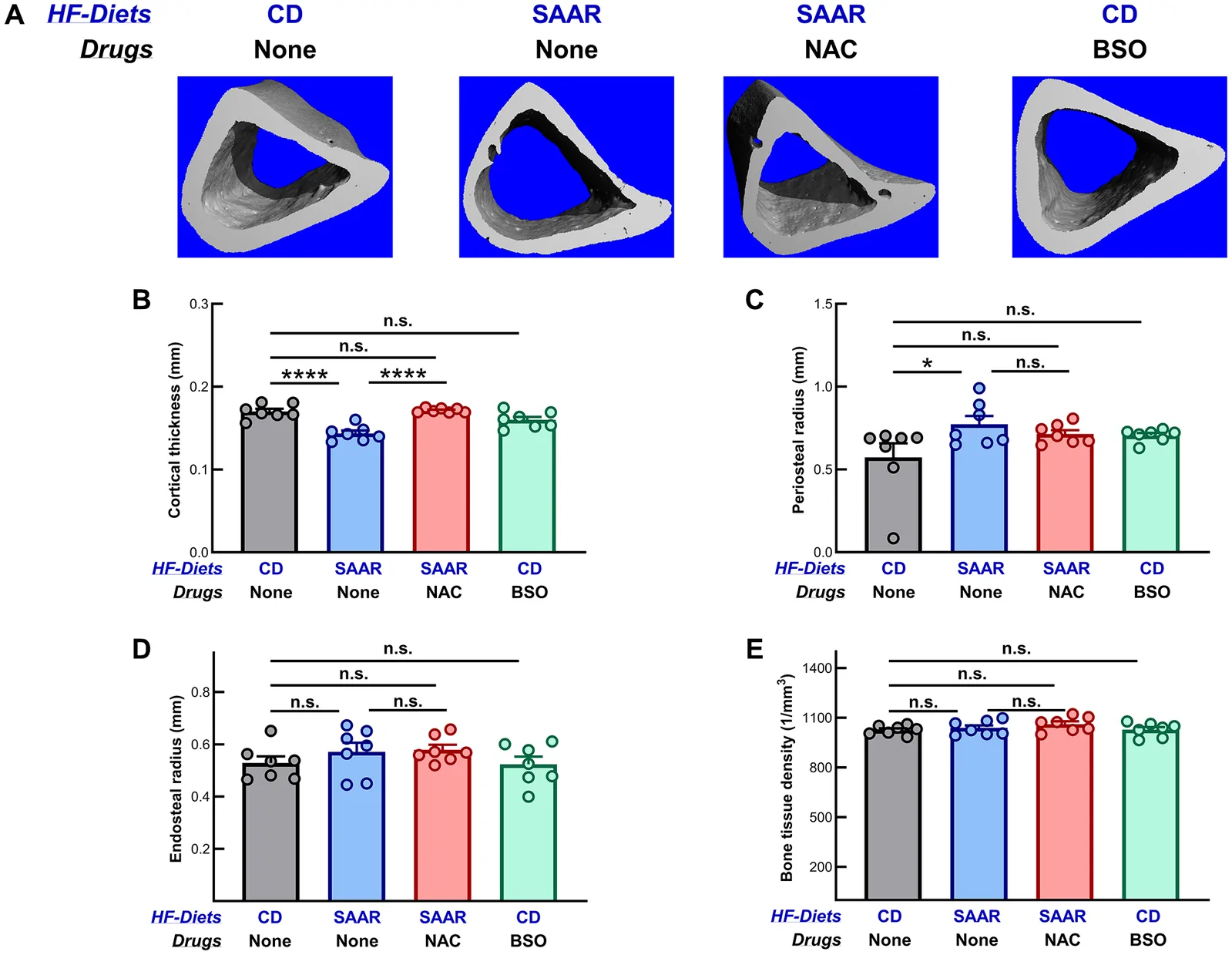
**Unlike the SAAR diet, BSO does not decrease cortical bone mineral density.** Images of cortical cross-sections are shown in (**A**). Similar to its effect on the trabecular bone, the SAAR diet (**B**) decreased cortical thickness (SAAR < CD) and (**C**) increased periosteal radius (SAAR > CD), indicating a decrease in cortical bone density. However, the SAAR diet did not affect (**D**) endosteal radius (SAAR = CD) and (**E**) bone tissue density (SAAR = CD). NAC, as expected, reversed the decrease in cortical thickness (SAAR < [CD = NAC] in figure B) but did not affect other measures. BSO did not exert any effects at all (CD = BSO). Note: Sample sizes and statistical methods are similar to those in Figure 1.

### BSO did not affect osteoblast and osteoclast numbers, while the SAAR diet decreased both

A previous study in mice reported that the SAAR diet decreases bone mass and bone strength, along with a decrease in osteoblast proliferation [6]. The data from the current study on the effects of the SAAR diet on bone biomechanical strength and bone mass are consistent with those from the previous study [6]. In addition, we demonstrate that CysR mediates these undesired effects, but not MetR; the numbers of osteoblasts and osteoclasts in CD and NAC were similar. We quantified osteoblasts and osteoclasts using histomorphometry (Figure 3A). 5-micron thin sections were cut from fixed, non-decalcified plastic-embedded tibia and stained with toluidine blue. As can be seen in the control bones (CD), a large number of mature osteoblasts (dark blue-stained cells, indicated by red arrowheads) are in close juxtaposition to the bone surface. Quantified data show that the number of osteoblasts in SAAR mice was 31% of that in CD mice (Figure 3B, *P* = 0.0001). Notably, NAC reversed this decrease, as evidenced by an osteoblast frequency, which was 86% of that in CD mice (Figure 3B, *P* > 0.05). Osteocalcin is a well-recognized circulating marker of bone formation that reflects osteoblast activity. The mean plasma osteocalcin concentration was slightly lower (82%) in the SAAR group compared to the CD group, but it was not statistically significant (Supplementary Figure 2). In the NAC group, osteocalcin concentrations were 189% of those in the SAAR group, indicating the ability of NAC to reverse the effects of the SAAR diet despite MetR (*P* = 0.02). Osteocalcin concentrations in the BSO group did not differ from those in any other group (Supplementary Figure 2). The SAAR diet also decreased the number of osteoclasts (52% of CD; P_2t_ = 0.01; *P*-value from post-hoc analysis was 0.068), but its effect was milder than the effect on osteoblasts (Figure 3C). NAC also tended to reverse the SAAR-diet-induced decrease in osteoclast number, lowering it to 72% of that in the CD mice (Figure 3C, *P* = 0.43). BSO did not affect osteoblast and osteoclast numbers.

**Figure 3:**
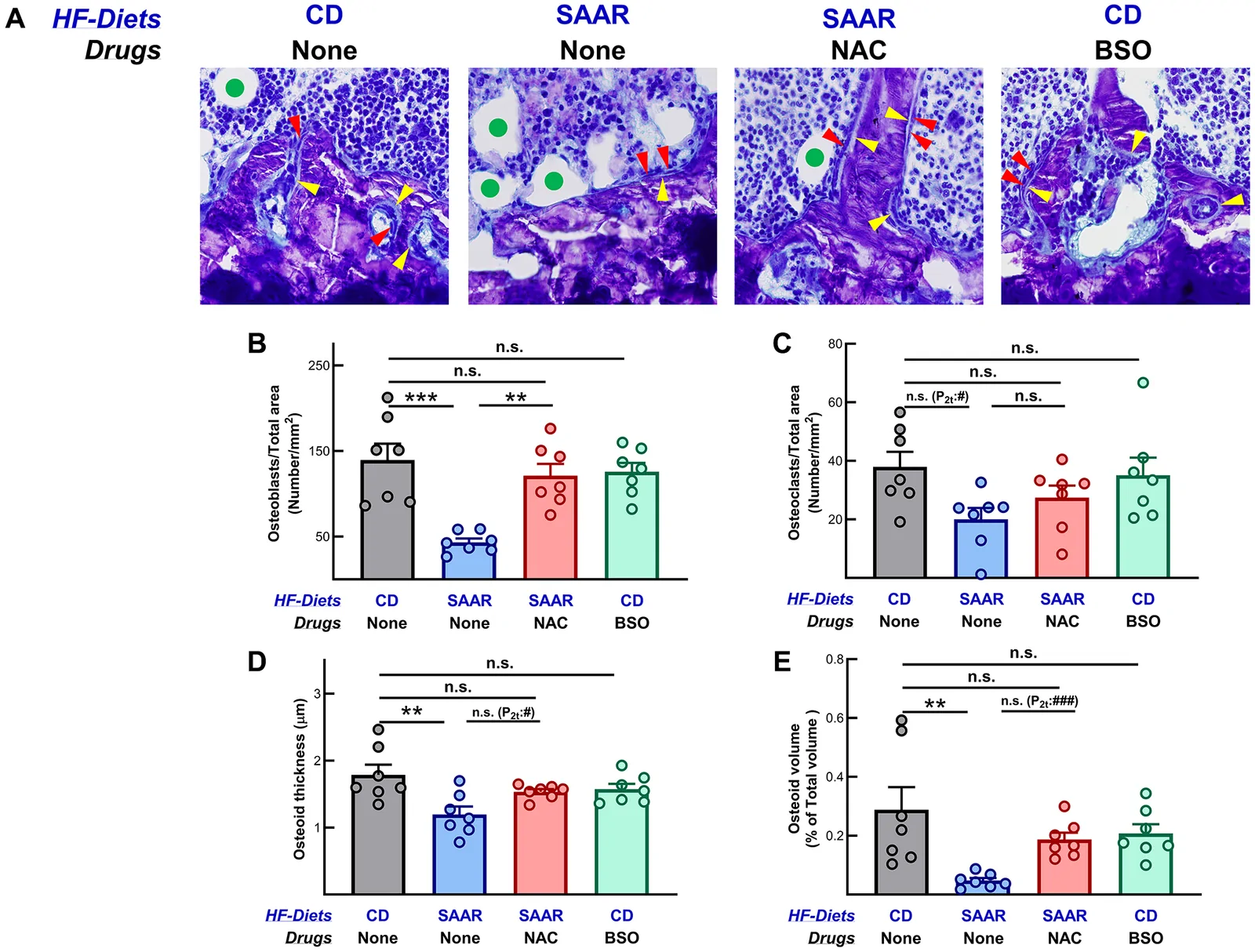
**The SAAR diet and BSO exert opposing effects on osteoblast number.** Photomicrographs of the proximal tibia (40X magnification) showing osteoblasts on bone surfaces (red arrowheads), osteoid (yellow arrowheads), and bone marrow adipocytes (green circles in the adipocytes) are shown in (**A**). Consistent with the changes in bone mineral density, the SAAR diet decreased the number of (**B**) osteoblasts (SAAR < CD) and (**C**) osteoclasts (SAAR < CD). NAC reversed the effects of the SAAR diet on osteoblasts ((SAAR < [CD = NAC]) but not on osteoclasts (SAAR = NAC = CD). The SAAR diet also decreased (**D**) osteoid thickness and (**E**) osteoid volume; NAC reversed both. BSO did not affect any of these measures. Note: Sample sizes and statistical methods are similar to those in Figure 1.

The changes induced by the SAAR diet and NAC in osteoid thickness paralleled the changes they caused in osteoblast abundance (Figure 3D). In SAAR mice, the osteoid thickness was 66% of that in the CD mice (*P* = 0.003), while in the NAC mice, it was 85% (Figure 3D). In CD mice, the prominent seam of osteoid (light blue staining, yellow arrowheads in Figure 3A) secreted onto the bone surfaces indicates that osteoblasts are active. A strikingly different picture is seen from the bones of the SAAR-fed mice. Few osteoblasts and little osteoid is seen on bone surfaces, especially when marrow adipocytes (green circles) are close to bone surfaces. In contrast, similar to those in the CD mice, NAC mice had a higher number of osteoblasts and more osteoid on bone surfaces. These changes indicate that NAC corrected the loss of osteoblasts and osteoid volume caused by SAAR (Figure 3A, 3B, 3E). Histologically, the BSO mice were not different from the CD mice.

### Mice on BSO had bones with similar mechanical strength to those on the control diet

In the SAAR mice, indicators of bone strength, including femur stiffness and maximum load, were 64% and 70% of those in the CD group (Figure 4A, 4B). Feeding mice the same SAAR diet, while supplementing NAC in their water, reversed these values to those observed in mice on the CD diet. Of note, although neither the SAAR diet nor BSO had significant effects on the total work, compared to CD, the direction of the change was opposite, i.e., in the SAAR group, the average total work tended to be lower, while in the BSO group it tended to be higher (Figure 4C). An unpaired two-tailed *t*-test between the SAAR diet and BSO indicates a significant difference between the two groups (P_2t_ = 0.004).

**Figure 4:**
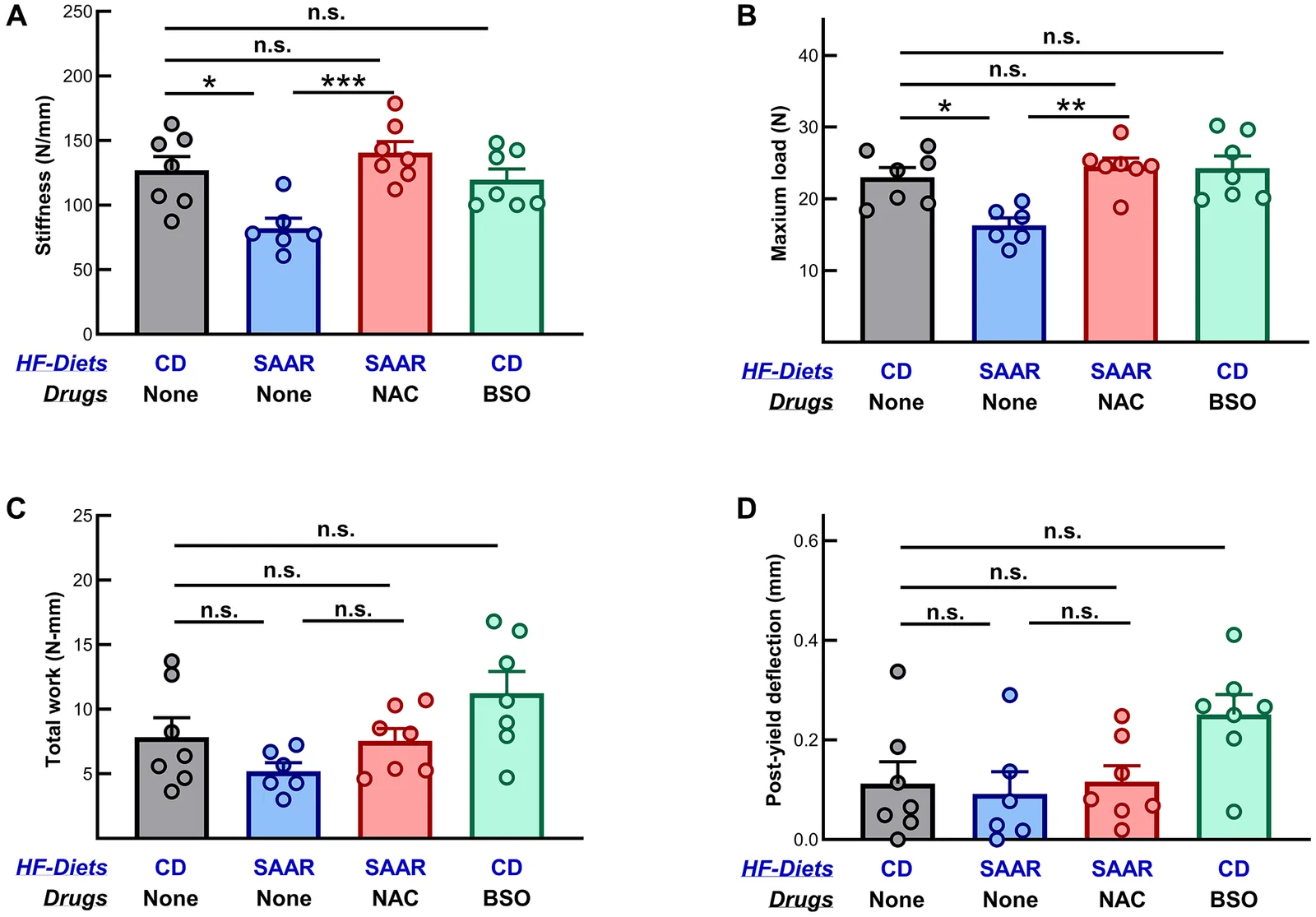
**Mice on the BSO had bones with similar mechanical strength to those on the control diet.** The SAAR diet decreased (**A**) stiffness (SAAR < CD) and (**B**) maximum load (SAAR < CD), while NAC reversed both effects (SAAR < [CD = NAC]). Neither the SAAR diet nor NAC affected (**C**) total work (SAAR = CD = NAC) and (**D**) post-yield deflection (SAAR = CD = NAC). BSO did not affect any measure of mechanical strength at all (CD = BSO). Note: Sample sizes and statistical methods are similar to those in Figure 1.

Post-yield deflection was similar among the CD, the SAAR diet, and the NAC groups (Figure 4D). The BSO group tended to have higher post-yield deflection than all three groups. While no differences were observed in post-hoc group-wise comparisons, *t*-tests revealed that the BSO group was statistically different from both the CD (P_2t_ = 0.02) and the SAAR (P_2t_ = 0.01) groups.

### The SAAR diet, but not BSO, increases marrow adipocytes

Unlike its effects on other body fat depots, the SAAR diet increases bone marrow adipogenesis [6]. To investigate the effects of NAC and BSO supplementation on marrow adipocytes, micro-CT analysis of decalcified and osmium-stained tibiae was conducted. Marrow adipocytes were quantified at four volumes of interest (epiphyseal [above the growth plate, VOI-1], proximal metaphysis [just below the growth plate, VOI-2], diaphysis [VOI-3], and distal diaphysis [VOI-4], Figure 5A). VOI-2 and VOI-4 in the SAAR group were 253% (*P* = 0.007) and 270% (*P* = 0.02) of CD, respectively (Figure 5B, 5D). Although VOI-1 (123% of CD, *P* = 0.62) and VOI-3 (187% of CD, *P* = 0.15) were higher in SAAR than CD, the differences were not statistically significant (Figure 5C, data for VOI-1 are not shown). A previous study reported a robust increase in the marrow adipocytes in all VOIs by the SAAR diet [7]. Differences in the experimental design, including the age at which the intervention was started (five weeks versus eighteen weeks), strain of the mice (C56BL6/J and C56BL6/NTac), and dietary fat content (10% Kcal from fat versus 60% Kcal from fat; dietary fat content in the previous study is inferred from the body weights) might contribute to these differences.

**Figure 5:**
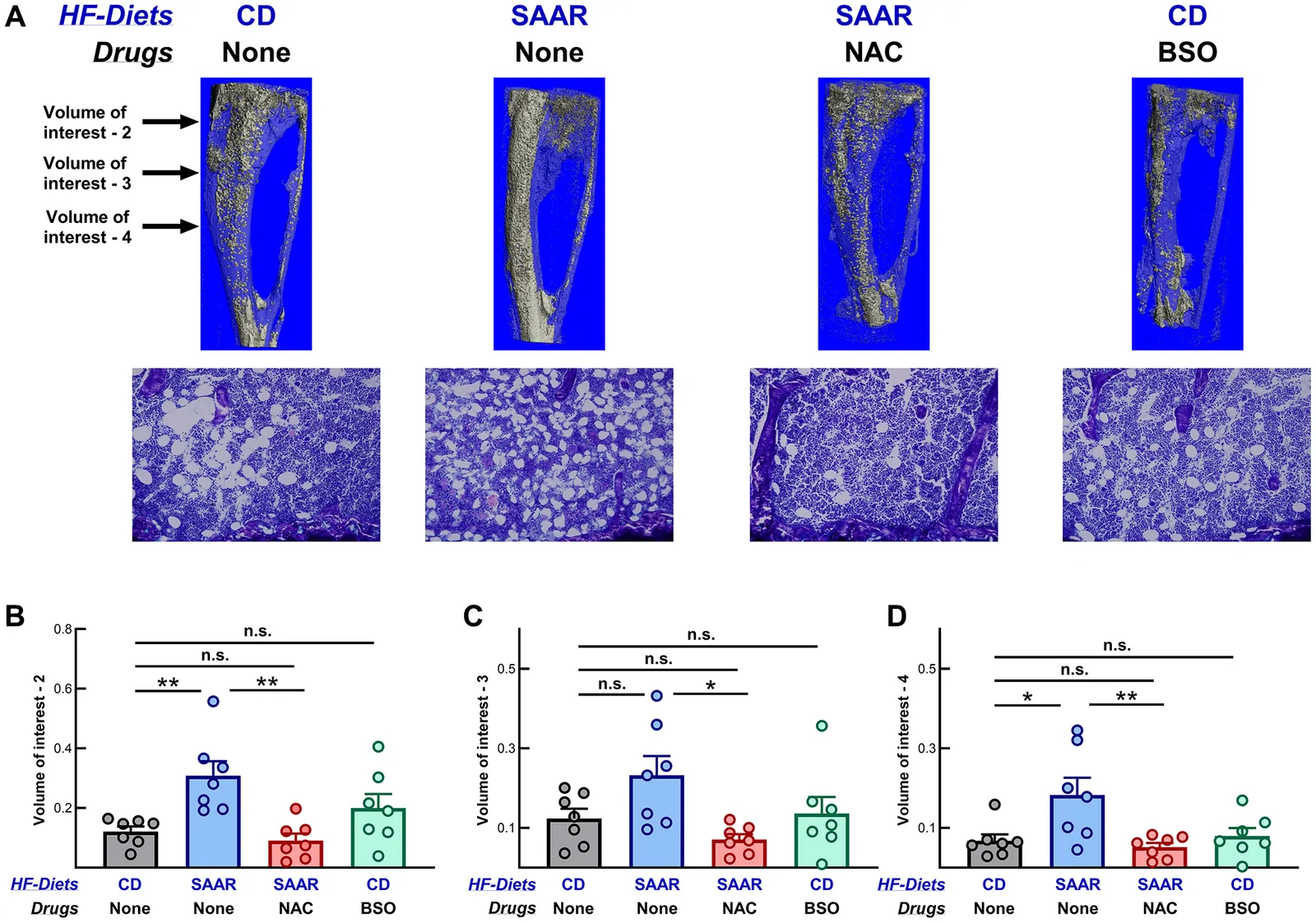
**The SAAR diet, but not BSO, increases marrow adipocytes in tibiae.** Micro-CT (first row) and photomicrographs (second row) of osmium-stained images of tibiae are shown in (A). Compared with the CD, the SAAR diet increased marrow adipocytes at volumes of interest 2 and 4, but not at volume of interest 3 (**B**–**D**, SAAR > CD). NAC reversed the increase in SAAR at all volumes of interest (**B**–**D**, SAAR > [CD = NAC]), while BSO had no effect (CD = BSO). Sample sizes and statistical methods are similar to those in Figure 1. Note: Volume of interest-1 is not shown in the figure.

Despite feeding on the SAAR diet, marrow adipocyte numbers at all VOIs of mice in the NAC group were similar to those in the CD group, suggesting that CysR mediates the effects of the SAAR diet on marrow adipocytes, but not MetR (Figure 5). Although BSO was able to decrease the total body fat and epididymal fat weights, it did not exert any appreciable effects on marrow adipocytes (Figure 5 and Supplementary Figure 1).

## DISCUSSION

Multiple studies have shown that the lifespan- and healthspan-extending SAAR diet exerts detrimental effects on bone health; however, the underlying mechanisms remain unclear [6, 7, 20]. Due to the lack of Cys in the SAAR diet, animals undergo CysR in addition to MetR. We previously reported that the SAAR diet-induced changes in the plasma concentrations of IGF-1, FGF-21, leptin, and adiponectin are exclusively associated with either MetR or CysR [18]. In a follow-up study, we demonstrated that the anti-obesity effect of the SAAR diet was solely due to CysR; the SAAR diet lost its anti-obesity effect when CysR was eliminated by supplementing NAC [9]. In the current study, we investigated whether CysR is specifically associated with the detrimental effects of the SAAR diet on bone health. Data indicate that the SAAR diet-induced increase in marrow adipocytes, as well as the decreases in bone mineral density, mechanical strength, and the number of osteoblasts and osteoclasts, are all due to CysR, but not MetR. This finding has substantial implications for understanding the regulation of bone health by nutrients, particularly the effects of the sulfur-containing amino acids Met and Cys. Our data also suggest that, similar to the SAAR diet, non-toxic doses of BSO exert anti-adiposity effects without any adverse effects on bone health. Given the challenges associated with the long-term adherence to and formulation of the SAAR diet for human consumption, the selective effects of BSO on adipose metabolism make it a potential alternative.

Osteoblasts and marrow adipocytes arise from a common skeletal progenitor cell(s) in the bone marrow [21, 22]. Changes in the differentiation pathway of these cells can have a reciprocal relationship with their numbers. Thus, an increase in marrow adipocytes leads to a decrease in osteoblasts, resulting in a concomitant decrease in BMD and bone strength. Our data show that SAAR induces a marked increase in marrow adipogenesis, which is accompanied by a loss of BMD. Importantly, treatment of the SAAR mice with NAC prevents the increase in marrow adipogenesis throughout the medullary canals in tibiae and femurs. This inhibition of SAAR-induced marrow adipogenesis was accompanied by an increase in the number of osteoblasts, an increase in BMD, and an increase in bone strength. Overall, our study provides unequivocal evidence that it is CysR, not MetR, which mediates the detrimental effects of the SAAR diet on bone.

There is substantial evidence for the role of NAC in bone health. By altering *Wnt5a* and *Ppar-γ* expression, NAC favored the differentiation of bone marrow stromal cells into osteoblasts while simultaneously inhibiting their differentiation into adipogenic cells [23]. Other studies report that NAC attenuates osteoclast differentiation, induces osteoblast differentiation, and increases mineralization of bone matrix [24, 25]. These data are consistent with our findings of an increase in the number of osteoblasts in the NAC group compared to the SAAR group (Figure 3B). The functional consequences of increased osteoblast number were also evident, as NAC mice had higher bone mineral density and mechanical strength than SAAR mice (Figures 1, 2 and 4). However, the mechanistic effects of NAC on marrow adipocytes have not been studied well. One *in vitro* study reported that NAC decreases the accumulation of triglycerides and increases β-oxidation in bone marrow adipocytes [26]. While we did not quantify the bone marrow triglyceride concentrations, we speculate that NAC might be inducing similar changes in our study. Our observation of fewer marrow adipocytes in the NAC group than in the SAAR group aligns well with the effects of NAC on triglycerides and β-oxidation reported in other studies (Figure 5).

NAC can affect bone health through its own biochemical properties or by replenishing cysteine and glutathione in tissues. Cys- and GSH-dependent mechanisms include regulation of *Scd-1* expression, modulation of cellular antioxidant capacity, and the effects on body weight. Studies show that NAC, by increasing plasma Cys, reversed the SAAR diet-induced increase in *Scd-1* expression in white adipose tissue [27]. Although we did not quantify *Scd-1* expression, considering its contribution to osteoblast growth, a similar reversal remains a potential mechanism [28, 29]. Optimal GSH concentrations, required to prevent oxidative stress, are essential for maintaining the balance between osteoblasts and osteoclasts [30, 31]. Both animal and human studies highlight the role of mechanical load imposed by increased body weight in maintaining bone health, with some linking higher plasma Cys levels to increased bone mineral density through an increase in fat mass [32–35]. In our study, SAAR mice lost 40% of their total body weight and 75% of their fat mass within four weeks, whereas those on NAC continued to gain weight (Supplementary Figure 1A, 1B). Such rapid weight loss, which also occurs in bariatric surgery and caloric restriction, can weaken bones. The lack of weight loss and the continued mechanical load on bones might contribute to the effects of NAC on bone [32, 34, 36].

NAC could directly affect bone health through its antioxidant function and ability to reduce disulfide bonds. Limited evidence suggests that NAC *per se* can exert antioxidant effects, as it can scavenge particular reactive oxygen species and aldehydes, potentially altering the osteoblast-to-osteoclast balance [37, 38]. The antioxidant function of NAC is highly relevant in the current study, as all mice were fed a high-fat diet, a significant source of reactive oxygen species. A high-fat diet was demonstrated to decrease the abundance of pluripotent lin^−^/c-Kit^+^ cells in bone marrow [39]. This effect was reversed by either the overexpression of antioxidant enzymes, including superoxide dismutase 1 and 3, and glutathione peroxidase, or by NAC supplementation, strongly suggesting that a high-fat diet affects osteoblast numbers [24, 25, 39]. Thus, whether supplementing the SAAR diet with NAC on a 10% fat diet would yield similar findings as in the current study should be investigated in future studies. Because NAC is a stronger nucleophile than Cys and GSH, it is a more potent disulfide bond reductant [37]. Functionality of many molecules associated with bone formation and modeling, including osteoprotegerin, RANKL, and procollagen, is susceptible to disulfide bond reduction [40–42]. While additional studies are required to confirm which of these mechanisms play a causal role, determining this will be challenging.

In the parent study, to determine if CysR mediates the anti-obesity phenotype of the SAAR diet by lowering GSH levels, we included a BSO group. We reported a potent anti-adiposity effect of BSO on epididymal fat mass, total body fat mass, and liver lipid droplet frequency, all of which were significantly lower than those in the CD group [9]. Despite such strong effects, unlike the SAAR diet, BSO did not induce any detrimental effects on bone (Figures 1–5).

The lack of an effect of BSO on bone health is intriguing from a mechanistic perspective. One previous study reported the anti-adiposity effects of BSO in C3H/HeH mice but did not present any data on bone health [43]. However, this study reported tissue-specific effects of BSO on Cys and GSH concentrations. We observed similar tissue-specific effects in our parent study, a plausible reason for the lack of effects on bone [9]. Although BSO was very effective in decreasing hepatic and renal GSH in our parent study, the decrease in renal GSH was much higher (39% of CD) than in hepatic GSH (60% of CD) [9]. BSO might exert even milder effects on the GSH in bone marrow than in the liver. One previous study shows that bone marrow was the most resistant tissue to GSH depletion by BSO [44]. Another study reported a biphasic effect of BSO on the *in vitro* differentiation of osteoclast-like RAW cells. BSO increased the differentiation of these cells at concentrations ranging from 1 μM to 3 μM, whereas it had the opposite effect at concentrations from 3 μM to 20 μM [24]. Our data show no significant effect of BSO on osteoclast number, probably due to unaltered bone marrow GSH. Other studies report that osteoclast proliferation depends not only on total GSH levels, but also on the ratio of oxidized-to-reduced GSH, which can be altered by dietary fat content [45, 46]. Due to the lack of data on the total levels and the ratio of oxidized-to-reduced GSH in the bone marrow, we cannot determine a specific reason for the lack of effect of BSO on the bones. Additional studies are required to draw such conclusions.

One valid concern regarding the use of BSO as a therapeutic agent is its toxicity and oxidative stress as a result of severe GSH depletion. We earlier reported that when used at appropriate concentrations, BSO improved health in mice without exerting toxicity. When administered in water at 15 mM with *ad libitum* access, BSO improved glucose tolerance in Akita mice, which are prone to type-1b diabetes [47]. We also reported the anti-obesity effect of BSO in mice on a 60% Kcal fat diet, when administered in water at 30 mM [9]. In either of these studies, BSO did not increase plasma markers of either liver damage or kidney damage, including aspartate transaminase, alanine transaminase, and cystatin-C. There is also growing evidence that excess GSH concentrations induce reductive stress, eventually leading to metabolic diseases and cancers [48, 49]. Limiting GSH synthesis in genetically modified mice has also been reported to ameliorate triglyceride synthesis without inducing hepatic failure [50]. Based on these data, we propose that careful consideration of low-dose BSO to inhibit GSH biosynthesis in targeted organs and specific health conditions might aid in improving human health. However, except for its use in cancers, data on the clinical use of BSO are limited. When used in combination with chemotherapy and radiotherapy, preclinical and clinical studies show that BSO was synergistic against cancers [51–56]. Long-term preclinical studies are necessary to understand the off-target effects on organs susceptible to oxidative stress, including the brain, and to investigate any sexually dimorphic effects, to establish the safety of BSO as a therapeutic agent. We are not aware of whether clinical-grade BSO is commercially available. However, previous studies in humans mentioned the use of clinical-grade BSO, provided by the National Cancer Institute, indicating that manufacturing clinical-grade BSO is feasible [51, 54–56].

Our understanding of the dietary regulation of bone health in the context of micronutrient deficiencies, such as calcium, vitamins A and D, and macronutrient deficiencies, including protein, has steadily improved over the decades [57–59]. New dietary patterns, including caloric restriction, SAAR, ketogenic diets, and time-restricted eating, are being investigated for their potential to extend lifespan and healthspan in laboratory models. Although a few clinical studies have been conducted with some of these diets, long-term studies are relatively rare. Preclinical studies, however, suggest that the effect of these diets on bone health is variable, with most of the studies reporting deleterious effects and a few reporting a modest benefit or no effect at all [7, 22, 60–64]. Identifying avenues to avoid the adverse effects on bone is essential for successfully translating these interventions. For instance, metformin was reported to attenuate the adverse effects caused by the ketogenic diet on bone, suggesting that the combinatorial intervention might be more efficacious [65]. In a previous study, we reported that a modified SAAR regimen, which involves feeding a control diet for four days a week and a Met-free diet (0% Met) for three days a week, was less deleterious to bones while providing the same healthspan extension benefits as the continuous regimen [6]. From a translational perspective, formulating Met-free diets for human consumption would be challenging. BSO supplementation, without altering dietary formulations or regimens, offers a more practical way to achieve the same benefits as the SAAR diet without the associated adverse effects on bones. Alternatively, a pharmacodietary approach, i.e., combining the SAAR diet intervention with BSO treatment, might be more efficacious. The ability of the BSO to prevent weight gain without negatively impacting bones, along with the SAAR diet’s capacity to induce a lean phenotype in obese mice despite its adverse effect on bones, suggests that these two interventions might activate unique mechanisms, indicating a potential synergism between them. If these two interventions synergize, simultaneous treatment with BSO at a dose lower than 30 mM, combined with feeding a modified version of the SAAR diet with total sulfur amino acid concentrations between 0.12% and 0.86%, would minimize the toxicity of BSO and the negative impact of the SAAR diet on bones.

We recognize that our study has some limitations. It has yet to be determined whether BSO would exert the same benefits on body composition and bone health in female mice as in male mice. The availability of more literature on using BSO in males compared to females prompted us to conduct our studies in males. A few published papers, however, report that female mice are more sensitive to BSO than males [66]. We are conducting comparative studies in males and females to find an effective therapeutic dose in females. Because the primary objective of our parent study was to investigate whether BSO exerts anti-obesity effects, we formulated our diets with 60% of calories from fat. Considering that high-fat diets induce marrow adipogenesis, additional studies are required to confirm similar effects of BSO in mice on diets with normal fat content [67]. While we collected as much data as possible to test our hypothesis, biochemical data from the bone marrow, such as GSH and Cys levels, the expression of genes associated with proliferation, and the activities of osteoblasts and osteoclasts, would have helped gain more mechanistic insights. Overall, our study provides the first evidence for the role of CysR in the SAAR diet-induced adverse effects on bone health, and that BSO induces beneficial changes in body composition without any adverse effects on bones. These data lay the foundation for interdisciplinary studies in nutrition, aging, and orthopedics.

## Supplementary Materials

Supplementary Figures

## References

[1] Howard AC, Mir D, Snow S, Horrocks J, Sayed H, Ma Z, Rogers AN. Anabolic Function Downstream of TOR Controls Trade-offs Between Longevity and Reproduction at the Level of Specific Tissues *in C. elegans*. Front Aging. 2021; 2:725068. 10.3389/fragi.2021.725068 35340273 PMC8953723

[2] Imai S. SIRT1 and caloric restriction: an insight into possible trade-offs between robustness and frailty. Curr Opin Clin Nutr Metab Care. 2009; 12:350–6. 10.1097/MCO.0b013e32832c932d 19474721 PMC2734391

[3] Nichenametla SN, Mattocks DAL, Malloy VL. Age-at-onset-dependent effects of sulfur amino acid restriction on markers of growth and stress in male F344 rats. Aging Cell. 2020; 19:e13177. 10.1111/acel.13177 32573078 PMC7426777

[4] Ables GP, Perrone CE, Orentreich D, Orentreich N. Methionine-restricted C57BL/6J mice are resistant to diet-induced obesity and insulin resistance but have low bone density. PLoS One. 2012; 7:e51357. 10.1371/journal.pone.0051357 23236485 PMC3518083

[5] Cooke D, Mattocks D, Nichenametla SN, Anunciado-Koza RP, Koza RA, Ables GP. Weight Loss and Concomitant Adipose Autophagy in Methionine-Restricted Obese Mice is Not Dependent on Adiponectin or FGF21. Obesity (Silver Spring). 2020; 28:1075–85. 10.1002/oby.22763 32348021 PMC7245564

[6] Plummer JD, Horowitz MC, Johnson JE. Intermittent Methionine Restriction Reduces Marrow Fat Accumulation and Preserves More Bone Mass than Continuous Methionine Restriction. Aging Biol. 2024; 2:20230019. 10.59368/agingbio.20230019 38550776 PMC10977905

[7] Plummer J, Park M, Perodin F, Horowitz MC, Hens JR. Methionine-Restricted Diet Increases miRNAs That Can Target RUNX2 Expression and Alters Bone Structure in Young Mice. J Cell Biochem. 2017; 118:31–42. 10.1002/jcb.25604 27191548 PMC5426510

[8] Nichenametla SN, Mattocks DAL, Malloy VL, Pinto JT. Sulfur amino acid restriction-induced changes in redox-sensitive proteins are associated with slow protein synthesis rates. Ann N Y Acad Sci. 2018; 1418:80–94. 10.1111/nyas.13556 29377163

[9] Ommi NB, Mattocks DAL, Kalecký K, Bottiglieri T, Nichenametla SN. Pharmacological recapitulation of the lean phenotype induced by the lifespan-extending sulfur amino acid-restricted diet. Aging (Albany NY). 2025; 17:960–81. 10.18632/aging.206237 40202448 PMC12074818

[10] Di Giacomo C, Malfa GA, Tomasello B, Bianchi S, Acquaviva R. Natural Compounds and Glutathione: Beyond Mere Antioxidants. Antioxidants (Basel). 2023; 12:1445. 10.3390/antiox12071445 37507985 PMC10376414

[11] Franco R, Cidlowski JA. Apoptosis and glutathione: beyond an antioxidant. Cell Death Differ. 2009; 16:1303–14. 10.1038/cdd.2009.107 19662025

[12] Fujita H, Ochi M, Ono M, Aoyama E, Ogino T, Kondo Y, Ohuchi H. Glutathione accelerates osteoclast differentiation and inflammatory bone destruction. Free Radic Res. 2019; 53:226–36. 10.1080/10715762.2018.1563782 30741054

[13] Hu G, Yu Y, Sharma D, Pruett-Miller SM, Ren Y, Zhang GF, Karner CM. Glutathione limits RUNX2 oxidation and degradation to regulate bone formation. JCI Insight. 2023; 8:e166888. 10.1172/jci.insight.166888 37432749 PMC10543723

[14] Lean JM, Davies JT, Fuller K, Jagger CJ, Kirstein B, Partington GA, Urry ZL, Chambers TJ. A crucial role for thiol antioxidants in estrogen-deficiency bone loss. J Clin Invest. 2003; 112:915–23. 10.1172/JCI18859 12975476 PMC193670

[15] Horowitz MC, Xi Y, Pflugh DL, Hesslein DG, Schatz DG, Lorenzo JA, Bothwell AL. Pax5-deficient mice exhibit early onset osteopenia with increased osteoclast progenitors. J Immunol. 2004; 173:6583–91. 10.4049/jimmunol.173.11.6583 15557148

[16] Parfitt AM, Drezner MK, Glorieux FH, Kanis JA, Malluche H, Meunier PJ, Ott SM, Recker RR. Bone histomorphometry: standardization of nomenclature, symbols, and units. Report of the ASBMR Histomorphometry Nomenclature Committee. J Bone Miner Res. 1987; 2:595–610. 10.1002/jbmr.5650020617 3455637

[17] Scheller EL, Troiano N, Vanhoutan JN, Bouxsein MA, Fretz JA, Xi Y, Nelson T, Katz G, Berry R, Church CD, Doucette CR, Rodeheffer MS, Macdougald OA, et al. Use of osmium tetroxide staining with microcomputerized tomography to visualize and quantify bone marrow adipose tissue in vivo. Methods Enzymol. 2014; 537:123–39. 10.1016/B978-0-12-411619-1.00007-0 24480344 PMC4097010

[18] Nichenametla SN, Mattocks DAL, Cooke D, Midya V, Malloy VL, Mansilla W, Øvrebø B, Turner C, Bastani NE, Sokolová J, Pavlíková M, Richie JP Jr, Shoveller AK, et al. Cysteine restriction-specific effects of sulfur amino acid restriction on lipid metabolism. Aging Cell. 2022; 21:e13739. 10.1111/acel.13739 36403077 PMC9741510

[19] Elshorbagy AK, Valdivia-Garcia M, Mattocks DA, Plummer JD, Smith AD, Drevon CA, Refsum H, Perrone CE. Cysteine supplementation reverses methionine restriction effects on rat adiposity: significance of stearoyl-coenzyme A desaturase. J Lipid Res. 2011; 52:104–12. 10.1194/jlr.M010215 20871132 PMC2999932

[20] Huang TH, Lewis JL, Lin HS, Kuo LT, Mao SW, Tai YS, Chang MS, Ables GP, Perrone CE, Yang RS. A methionine-restricted diet and endurance exercise decrease bone mass and extrinsic strength but increase intrinsic strength in growing male rats. J Nutr. 2014; 144:621–30. 10.3945/jn.113.187922 24647387

[21] Pierce JL, Begun DL, Westendorf JJ, McGee-Lawrence ME. Defining osteoblast and adipocyte lineages in the bone marrow. Bone. 2019; 118:2–7. 10.1016/j.bone.2018.05.019 29782940 PMC6240509

[22] Rosen CJ, Horowitz MC. Nutrient regulation of bone marrow adipose tissue: skeletal implications of weight loss. Nat Rev Endocrinol. 2023; 19:626–38. 10.1038/s41574-023-00879-4 37587198 PMC10592027

[23] Ji H, Liu Y, Zhao X, Zhang M. N-acetyl-L-cysteine enhances the osteogenic differentiation and inhibits the adipogenic differentiation through up regulation of Wnt 5a and down regulation of PPARG in bone marrow stromal cells. Biomed Pharmacother. 2011; 65:369–74. 10.1016/j.biopha.2011.04.020 21775089

[24] Cao JJ, Picklo MJ. N-acetylcysteine supplementation decreases osteoclast differentiation and increases bone mass in mice fed a high-fat diet. J Nutr. 2014; 144:289–96. 10.3945/jn.113.185397 24381219

[25] Yamada M, Tsukimura N, Ikeda T, Sugita Y, Att W, Kojima N, Kubo K, Ueno T, Sakurai K, Ogawa T. N-acetyl cysteine as an osteogenesis-enhancing molecule for bone regeneration. Biomaterials. 2013; 34:6147–56. 10.1016/j.biomaterials.2013.04.064 23711675

[26] Raffaele M, Barbagallo I, Licari M, Carota G, Sferrazzo G, Spampinato M, Sorrenti V, Vanella L. N-Acetylcysteine (NAC) Ameliorates Lipid-Related Metabolic Dysfunction in Bone Marrow Stromal Cells-Derived Adipocytes. Evid Based Complement Alternat Med. 2018; 2018:5310961. 10.1155/2018/5310961 30416532 PMC6207898

[27] Elshorbagy AK, Valdivia-Garcia M, Mattocks DA, Plummer JD, Orentreich DS, Orentreich N, Refsum H, Perrone CE. Effect of taurine and N-acetylcysteine on methionine restriction-mediated adiposity resistance. Metabolism. 2013; 62:509–17. 10.1016/j.metabol.2012.10.005 23154184

[28] Tao J, Shi J, Lu Y, Dou B, Zhou Z, Gao M, Zhu Z. Overexpression of stearoyl-CoA desaturase 1 in bone-marrow mesenchymal stem cells increases osteogenesis. Panminerva Med. 2013; 55:283–9. 24088802

[29] Seo YJ, Park JH, Byun JH. Therapeutic Potential of Stearoyl-CoA Desaturase1 (SCD1) in Modulating the Effects of Fatty Acids on Osteoporosis. Cells. 2024; 13:1781. 10.3390/cells13211781 39513888 PMC11544805

[30] Basu S, Michaëlsson K, Olofsson H, Johansson S, Melhus H. Association between oxidative stress and bone mineral density. Biochem Biophys Res Commun. 2001; 288:275–9. 10.1006/bbrc.2001.5747 11594785

[31] Ezeriņa D, Takano Y, Hanaoka K, Urano Y, Dick TP. N-Acetyl Cysteine Functions as a Fast-Acting Antioxidant by Triggering Intracellular H_2_S and Sulfane Sulfur Production. Cell Chem Biol. 2018; 25:447–59.e4. 10.1016/j.chembiol.2018.01.011 29429900 PMC6455997

[32] Jensen VFH, Mølck AM, Dalgaard M, McGuigan FE, Akesson KE. Changes in bone mass associated with obesity and weight loss in humans: Applicability of animal models. Bone. 2021; 145:115781. 10.1016/j.bone.2020.115781 33285255

[33] Elshorbagy AK, Gjesdal CG, Nurk E, Tell GS, Ueland PM, Nygård O, Tverdal A, Vollset SE, Smith AD, Refsum H. Cysteine, homocysteine and bone mineral density: a role for body composition? Bone. 2009; 44:954–8. 10.1016/j.bone.2008.12.018 19168166

[34] Angrisani L, Santonicola A, Iovino P, Formisano G, Buchwald H, Scopinaro N. Bariatric Surgery Worldwide 2013. Obes Surg. 2015; 25:1822–32. 10.1007/s11695-015-1657-z 25835983

[35] Rinonapoli G, Pace V, Ruggiero C, Ceccarini P, Bisaccia M, Meccariello L, Caraffa A. Obesity and Bone: A Complex Relationship. Int J Mol Sci. 2021; 22:13662. 10.3390/ijms222413662 34948466 PMC8706946

[36] Liu L, Rosen CJ. New Insights into Calorie Restriction Induced Bone Loss. Endocrinol Metab (Seoul). 2023; 38:203–13. 10.3803/EnM.2023.1673 37150516 PMC10164494

[37] Zhitkovich A. *N*-Acetylcysteine: Antioxidant, Aldehyde Scavenger, and More. Chem Res Toxicol. 2019; 32:1318–9. 10.1021/acs.chemrestox.9b00152 31046246 PMC6757335

[38] Pedre B, Barayeu U, Ezeriņa D, Dick TP. The mechanism of action of N-acetylcysteine (NAC): The emerging role of H_2_S and sulfane sulfur species. Pharmacol Ther. 2021; 228:107916. 10.1016/j.pharmthera.2021.107916 34171332

[39] Xiao Y, Zhu Q, Liu X, Jiang M, Hao H, Zhu H, Cowan PJ, He X, Liu Q, Zhou S, Liu Z. High-fat diet selectively decreases bone marrow lin^-^ /CD117^+^ cell population in aging mice through increased ROS production. J Tissue Eng Regen Med. 2020; 14:884–92. 10.1002/term.3047 32337800 PMC7372732

[40] Nelson CA, Warren JT, Wang MW, Teitelbaum SL, Fremont DH. RANKL employs distinct binding modes to engage RANK and the osteoprotegerin decoy receptor. Structure. 2012; 20:1971–82. 10.1016/j.str.2012.08.030 23039992 PMC3607351

[41] Wang Y, Liu Y, Huang Z, Chen X, Zhang B. The roles of osteoprotegerin in cancer, far beyond a bone player. Cell Death Discov. 2022; 8:252. 10.1038/s41420-022-01042-0 35523775 PMC9076607

[42] Monson JM, Borstein P. Identification of a disulfide-linked procollagen as the biosynthetic precursor of chick-bone collagen. Proc Natl Acad Sci U S A. 1973; 70:3521–5. 10.1073/pnas.70.12.3521 4519643 PMC427272

[43] Elshorbagy AK, Jernerén F, Scudamore CL, McMurray F, Cater H, Hough T, Cox R, Refsum H. Exploring the Lean Phenotype of Glutathione-Depleted Mice: Thiol, Amino Acid and Fatty Acid Profiles. PLoS One. 2016; 11:e0163214. 10.1371/journal.pone.0163214 27788147 PMC5082875

[44] Kramer RA, Greene K, Ahmad S, Vistica DT. Chemosensitization of L-phenylalanine mustard by the thiol-modulating agent buthionine sulfoximine. Cancer Res. 1987; 47:1593–7. 3815359

[45] Hu G, Whitaker AL, Zhang GF, Karner CM. Divergent Requirements for Glutathione Biosynthesis During Osteoclast Differentiation In Vitro and In Vivo. Antioxidants (Basel). 2025; 14:197. 10.3390/antiox14020197 40002383 PMC11851466

[46] Li L, Zhang GF, Lee K, Lopez R, Previs SF, Willard B, McCullough A, Kasumov T. A Western diet induced NAFLD in LDLR(-/)(-) mice is associated with reduced hepatic glutathione synthesis. Free Radic Biol Med. 2016; 96:13–21. 10.1016/j.freeradbiomed.2016.03.032 27036364 PMC5297627

[47] Mattocks DAL, Ommi NB, Malloy VL, Nichenametla SN. An antireductant approach ameliorates misfolded proinsulin-induced hyperglycemia and glucose intolerance in male Akita mice. Geroscience. 2025; 47:1653–68. 10.1007/s11357-024-01326-6 39294474 PMC11979071

[48] Xiao W, Loscalzo J. Metabolic Responses to Reductive Stress. Antioxid Redox Signal. 2020; 32:1330–47. 10.1089/ars.2019.7803 31218894 PMC7247050

[49] Ji X, Xiao G. Reductive stress in cancer immunology and targeted therapy. Acta Biochim Biophys Sin (Shanghai). 2025. [Epub ahead of print]. 10.3724/abbs.2025173 40975792 PMC12862617

[50] Asantewaa G, Tuttle ET, Ward NP, Kang YP, Kim Y, Kavanagh ME, Girnius N, Chen Y, Duncan R, Rodriguez K, Hecht F, Zocchi M, Smorodintsev-Schiller L, et al. Glutathione supports lipid abundance *in vivo*. bioRxiv. 2023. 10.1101/2023.02.10.524960

[51] Tagde A, Singh H, Kang MH, Reynolds CP. The glutathione synthesis inhibitor buthionine sulfoximine synergistically enhanced melphalan activity against preclinical models of multiple myeloma. Blood Cancer J. 2014; 4:e229. 10.1038/bcj.2014.45 25036800 PMC4219442

[52] Hedley DW, McCulloch EA, Minden MD, Chow S, Curtis J. Antileukemic action of buthionine sulfoximine: evidence for an intrinsic death mechanism based on oxidative stress. Leukemia. 1998; 12:1545–52. 10.1038/sj.leu.2401138 9766498

[53] Dos Reis Oliveira C, Pereira JC, Barros Ibiapina A, Roseno Martins IR, de Castro E Sousa JM, Ferreira PMP, Carneiro da Silva FC. Buthionine sulfoximine and chemoresistance in cancer treatments: a systematic review with meta-analysis of preclinical studies. J Toxicol Environ Health B Crit Rev. 2023; 26:417–41. 10.1080/10937404.2023.2246876 37606035

[54] Anderson CP, Matthay KK, Perentesis JP, Neglia JP, Bailey HH, Villablanca JG, Groshen S, Hasenauer B, Maris JM, Seeger RC, Reynolds CP. Pilot study of intravenous melphalan combined with continuous infusion L-S,R-buthionine sulfoximine for children with recurrent neuroblastoma. Pediatr Blood Cancer. 2015; 62:1739–46. 10.1002/pbc.25594 26153194

[55] Bailey HH, Ripple G, Tutsch KD, Arzoomanian RZ, Alberti D, Feierabend C, Mahvi D, Schink J, Pomplun M, Mulcahy RT, Wilding G. Phase I study of continuous-infusion L-S,R-buthionine sulfoximine with intravenous melphalan. J Natl Cancer Inst. 1997; 89:1789–96. 10.1093/jnci/89.23.1789 9392620

[56] Bailey HH, Mulcahy RT, Tutsch KD, Arzoomanian RZ, Alberti D, Tombes MB, Wilding G, Pomplun M, Spriggs DR. Phase I clinical trial of intravenous L-buthionine sulfoximine and melphalan: an attempt at modulation of glutathione. J Clin Oncol. 1994; 12:194–205. 10.1200/JCO.1994.12.1.194 8270977

[57] Iguacel I, Miguel-Berges ML, Gómez-Bruton A, Moreno LA, Julián C. Veganism, vegetarianism, bone mineral density, and fracture risk: a systematic review and meta-analysis. Nutr Rev. 2019; 77:1–18. 10.1093/nutrit/nuy045 30376075

[58] Martiniakova M, Babikova M, Mondockova V, Blahova J, Kovacova V, Omelka R. The Role of Macronutrients, Micronutrients and Flavonoid Polyphenols in the Prevention and Treatment of Osteoporosis. Nutrients. 2022; 14:523. 10.3390/nu14030523 35276879 PMC8839902

[59] Karpouzos A, Diamantis E, Farmaki P, Savvanis S, Troupis T. Nutritional Aspects of Bone Health and Fracture Healing. J Osteoporos. 2017; 2017:4218472. 10.1155/2017/4218472 29464131 PMC5804294

[60] Zhu J, Liu C, Jia J, Zhang C, Yuan W, Leng H, Xu Y, Song C. Short-term caloric restriction induced bone loss in both axial and appendicular bones by increasing adiponectin. Ann N Y Acad Sci. 2020; 1474:47–60. 10.1111/nyas.14380 32469430

[61] Rinne C, Soultoukis GA, Oveisi M, Leer M, Schmidt-Bleek O, Burkhardt LM, Bucher CH, Moussa EA, Makhlouf M, Duda GN, Saraiva LR, Schmidt-Bleek K, Schulz TJ. Caloric restriction reduces trabecular bone loss during aging and improves bone marrow adipocyte endocrine function in male mice. Front Endocrinol (Lausanne). 2024; 15:1394263. 10.3389/fendo.2024.1394263 38904042 PMC11188307

[62] Wu X, Huang Z, Wang X, Fu Z, Liu J, Huang Z, Kong G, Xu X, Ding J, Zhu Q. Ketogenic Diet Compromises Both Cancellous and Cortical Bone Mass in Mice. Calcif Tissue Int. 2017; 101:412–21. 10.1007/s00223-017-0292-1 28547346

[63] Xu X, Ding J, Wu X, Huang Z, Kong G, Liu Q, Yang Z, Huang Z, Zhu Q. Bone microstructure and metabolism changes under the combined intervention of ketogenic diet with intermittent fasting: an in vivo study of rats. Exp Anim. 2019; 68:371–80. 10.1538/expanim.18-0084 30944267 PMC6699973

[64] Clayton DJ, Varley I, Papageorgiou M. Intermittent fasting and bone health: a bone of contention? Br J Nutr. 2023; 130:1487–99. 10.1017/S0007114523000545 36876592 PMC10551474

[65] Liu Q, Xu X, Yang Z, Liu Y, Wu X, Huang Z, Liu J, Huang Z, Kong G, Ding J, Li R, Lin J, Zhu Q. Metformin Alleviates the Bone Loss Induced by Ketogenic Diet: An In Vivo Study in Mice. Calcif Tissue Int. 2019; 104:59–69. 10.1007/s00223-018-0468-3 30167745

[66] Masubuchi Y, Nakayama J, Watanabe Y. Sex difference in susceptibility to acetaminophen hepatotoxicity is reversed by buthionine sulfoximine. Toxicology. 2011; 287:54–60. 10.1016/j.tox.2011.05.018 21672600

[67] Benova A, Tencerova M. Obesity-Induced Changes in Bone Marrow Homeostasis. Front Endocrinol (Lausanne). 2020; 11:294. 10.3389/fendo.2020.00294 32477271 PMC7235195

