# Supplementary material for: D, L-Buthionine-(S, R)-sulfoximine recapitulates the anti-obesity effects of sulfur amino acid restriction without the associated deleterious effects on bone in male mice
Source: Aging (Albany NY). 2026 Mar 1;18(1):82–99. doi: 10.18632/aging.206358 (PMC13632006; doi:10.18632/aging.206358)
Supplement: Supplementary file 1 [file aging-18-1-206358-s001.pdf]

SUPPLEMENTARY FIGURES

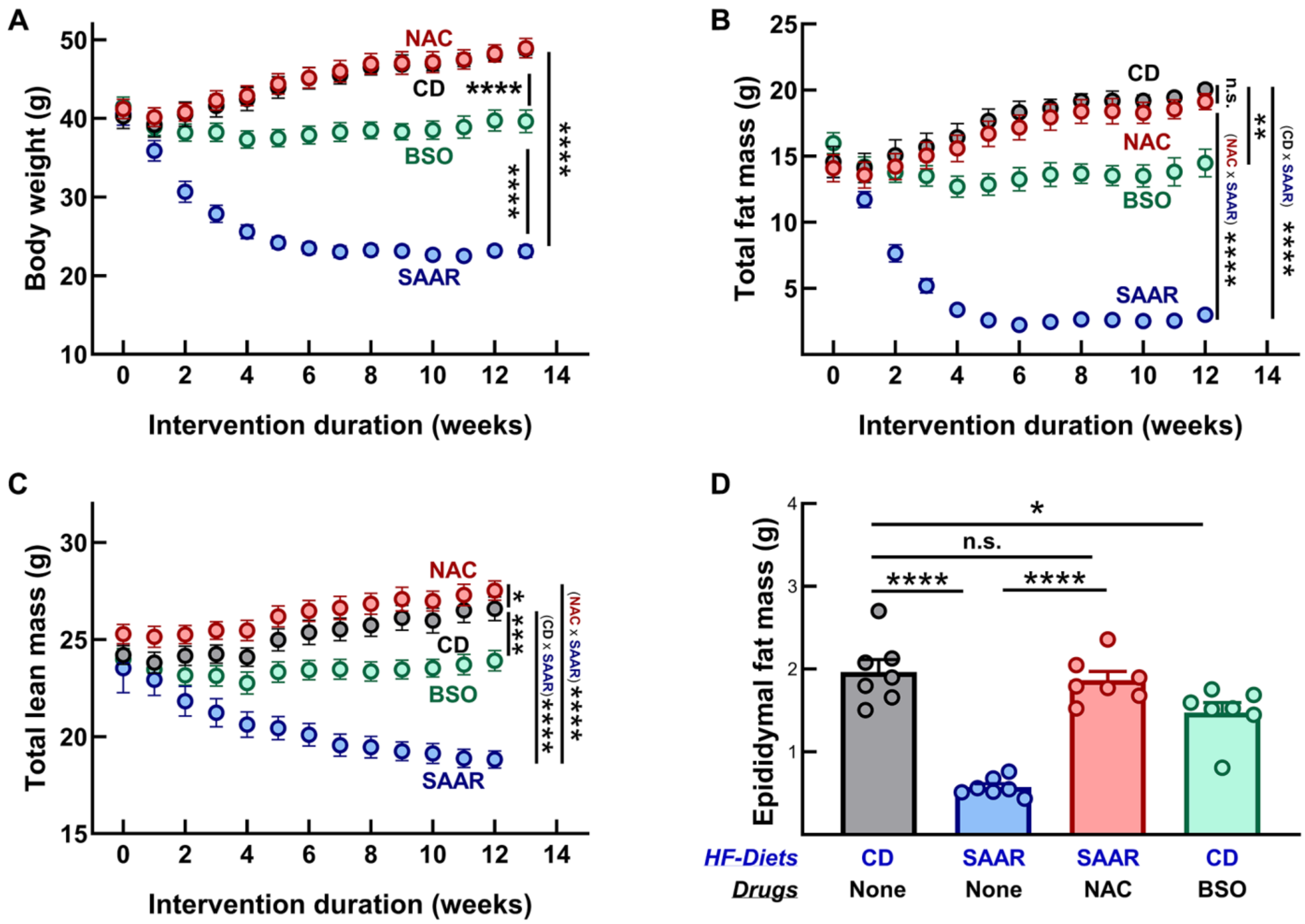

Supplementary Figure 1. Similar to the SAAR diet, BSO exerts anti-adiposity effects in mice on a high-fat diet. Compared to the CD, the SAAR diet decreased total body weight (**A**), fat mass (**B**), lean mass (**C**), and epididymal fat mass (**D**), i.e., SAAR < CD. NAC, reversed all these changes (**A**–**D**, SAAR < [CD = NAC]). Despite feeding on a high-fat diet replete with sulfur amino acids, mice in the BSO group had lower total body weight (**A**), fat mass (**B**), lean mass (**C**), and epididymal fat depot mass (**D**) than the CD mice, i.e., BSO < CD. Note: Sample sizes and statistical methods are similar to those in Figure 1. Bars and error bars represent means and standard errors of the mean. CD growth curves are invisible as NAC growth curves overlay them. The data were adapted from a previous publication that used the same animals as in the current study.

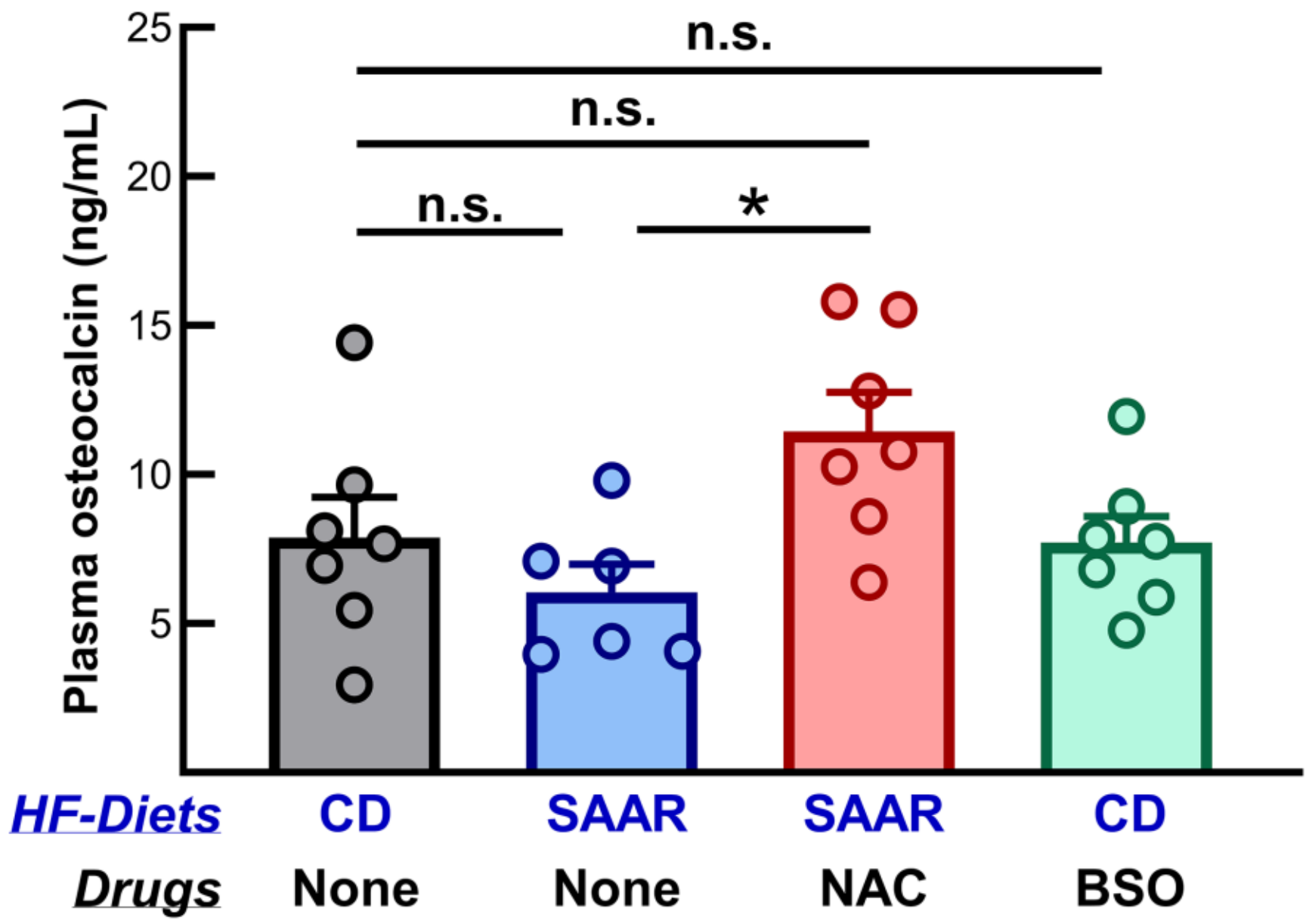

Supplementary Figure 2. NAC supplementation increases plasma osteocalcin concentrations. Mice on the SAAR diet had slightly lower concentrations of plasma osteocalcin than those on the CD diet ($P > 0.05$). NAC mice, despite feeding on the SAAR diet, had higher plasma osteocalcin concentrations (SAAR < NAC, $P < 0.05$). Note: Sample sizes and statistical methods are similar to those in Figure 1. Bars and error bars represent means and standard errors of the mean. CD growth curves are invisible as NAC growth curves overlay them. The data were adapted from a previous publication that used the same animals as in the current study.
